# A Semi-Analytical Solution for Elastic Analysis of Rotating Thick Cylindrical Shells with Variable Thickness Using Disk Form Multilayers

**DOI:** 10.1155/2014/932743

**Published:** 2014-02-17

**Authors:** Mohammad Zamani Nejad, Mehdi Jabbari, Mehdi Ghannad

**Affiliations:** ^1^Mechanical Engineering Department, Yasouj University, P.O. Box 75914-353, Yasouj, Iran; ^2^Mechanical Engineering Faculty, Shahrood University of Technology, Shahrood, Iran

## Abstract

Using disk form multilayers, a semi-analytical solution has been derived for determination of displacements and stresses in a rotating cylindrical shell with variable thickness under uniform pressure. The thick cylinder is divided into disk form layers form with their thickness corresponding to the thickness of the cylinder. Due to the existence of shear stress in the thick cylindrical shell with variable thickness, the equations governing disk layers are obtained based on first-order shear deformation theory (FSDT). These equations are in the form of a set of general differential equations. Given that the cylinder is divided into *n* disks, *n* sets of differential equations are obtained. The solution of this set of equations, applying the boundary conditions and continuity conditions between the layers, yields displacements and stresses. A numerical solution using finite element method (FEM) is also presented and good agreement was found.

## 1. Introduction

Thick cylindrical shells with variable thickness have widely been applied in many fields such as space fight, rocket, aviation, and submarine technology. Given the limitations of the classic theories of thick wall shells, very little attention has been paid to the analytical and semi-analytical solutions of these shells. Assuming the transverse shear effect, Naghdi and Cooper [1] formulated the theory of shear deformation. The solution of thick cylindrical shells of homogenous and isotropic materials using the first-order shear deformation theory (FSDT) was derived by Mirsky and Hermann [2]. Greenspon [3] opted to make a comparison between the findings regarding the different solutions obtained for cylindrical shells. Ziv and Perl [4] obtained the response of vibration analysis of a thick-walled cylindrical shell using FSDT theory and solved by finite difference method. Suzuki et al. [5] used the FSDT for vibration analysis of axisymmetric cylindrical shell with variable thickness. They assumed that the problem is in the state of plane stress and ignored the normal stress in the radial direction. Simkins [6] used the FSDT for determining displacement in a long and thick tube subjected to moving loads. A paper was also published by Kang and Leissa [7, 8] where equations of motion and energy functionals were derived for a three-dimensional coordinate system. The field equations are utilized to be expressed in terms of displacement components. Eipakchi et al. [9] used the FSDT for driving governing equations of thick cylinders with varying thickness and solved the equations with perturbation theory. Based on the FSDT and the virtual work principle, Ghannad et al. [10] obtained an elastic solution for thick truncated conical shells. Using tensor analysis, a complete 3-D set of field equations developed for elastic analysis of thick shells of revolution with arbitrary curvature and variable thickness along the meridional direction made of functionally graded materials (FGMs) by Nejad et al. [11]. Ghannad and Nejad [12] obtained the differential equations governing the homogenous and isotropic axisymmetric thick-walled cylinders with same boundary conditions at the two ends were generally derived, making use of first-order shear deformation theory and the virtual work principle. Following that, the set of nonhomogenous linear differential equations for the cylinder with clamped-clamped ends was solved. An analytical solution for clamped-clamped thick cylindrical shells with variable thickness subjected to constant internal pressure is presented by Ghannad et al. [13]. Using the first-order shear deformation theory and assuming the radially varying elastic modulus, Ghannad and Nejad [14] presented an analytical solution for displacements and stresses in pressurized thick heterogeneous cylindrical shells. Ghannad et al. [15] obtained an analytical solution for stresses and radial displacement for an FGM clamped-clamped pressurized thick cylindrical shell with variable thickness using shear deformation theory and matched asymptotic method.

In this paper, elastic analysis has been presented for rotating thick cylindrical shells under internal pressure with variable thickness using disk form multilayers.

## 2. Formulation of Problem

In the first-order shear deformation theory, the sections that are straight and perpendicular to the mid-plane remain straight but not necessarily perpendicular after deformation and loading. In this case, shear strain and shear stress are taken into consideration.

**Figure 1:**
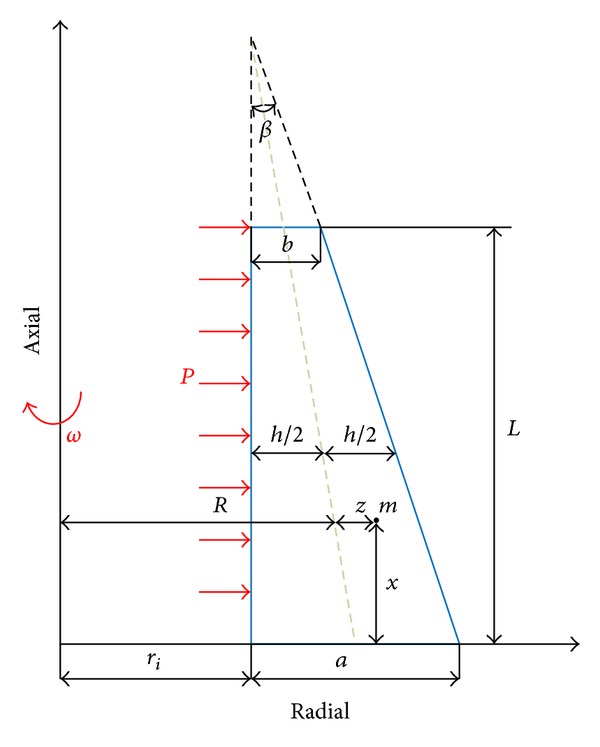
Thick cylindrical shell with variable thickness.

Geometry of a thick cylindrical shell with variable thickness *h*, and the length *L*, is shown in Figure 1.

The location of a typical point *m*, within the shell element is as

(1) $$\begin{array}{c} m:\left(r,x\right)=\left(R+z,x\right),\\ 0\leq x\leq L,\;\;-\frac{h}{2}\leq z\leq \frac{h}{2}, \end{array}$$

where *z* is the distance of typical point from the middle surface. In (1), *R* and variable thickness *h* are

(2) $$\begin{array}{c} R\left(x\right)=r_{i}+\frac{a}{2}-\frac{1}{2}\left(\tan\beta \right)x,\\ h\left(x\right)=r_{i}+a-\left(\tan\beta \right)x, \end{array}$$

where *β* is tapering angle as

(3) $$\begin{array}{c} \beta =\tan^{-1}\left(\frac{a-b}{L}\right). \end{array}$$

The general axisymmetric displacement field (*U*_*x*_, *U*_*z*_), in the first-order Mirsky-Hermann's theory [2], could be expressed on the basis of axial displacement and radial displacement as follows:

(4) $$\begin{array}{c} U_{x}\left(x,z\right)=u\left(x\right)+\phi \left(x\right)z,\\ U_{z}\left(x,z\right)=w\left(x\right)+\psi \left(x\right)z, \end{array}$$

where *u*(*x*) and *w*(*x*) are the displacement components of the middle surface. Also, *ϕ*(*x*) and *ψ*(*x*) are the functions used to determine the displacement field.

The kinematic equations (strain-displacement relations) in the cylindrical coordinates system are

(5) $$\begin{array}{c} \varepsilon_{x}=\frac{\partial U_{x}}{\partial x}=\frac{du}{dx}+\frac{d\phi }{dx}z,\\ \varepsilon_{\theta }=\frac{U_{z}}{r}=\left(\frac{w}{R+z}\right)+\left(\frac{\psi }{R+z}\right)z,\\ \varepsilon_{z}=\frac{\partial U_{z}}{\partial z}=\psi ,\\ \gamma_{xz}=\frac{\partial U_{x}}{\partial z}+\frac{\partial U_{z}}{\partial x}=\left(\phi +\frac{dw}{dx}\right)+\frac{d\psi }{dx}z. \end{array}$$

The stress-strain relations (constitutive equations) for homogeneous and isotropic materials are as follows:

(6) $$\begin{array}{c} \left\{\begin{array}{c} \sigma_{x}\\ \sigma_{\theta }\\ \sigma_{z}\\ \tau_{xz} \end{array}\right\}=\lambda \left[\begin{array}{cccc} 1-\upsilon & \upsilon & \upsilon & 0\\ \upsilon & 1-\upsilon & \upsilon & 0\\ \upsilon & \upsilon & 1-\upsilon & 0\\ 0 & 0 & 0 & \frac{1-2\upsilon }{2} \end{array}\right]\left\{\begin{array}{c} \varepsilon_{x}\\ \varepsilon_{\theta }\\ \varepsilon_{z}\\ \gamma_{xz} \end{array}\right\}, \end{array}$$

where *σ*_*i*_ and *ε*_*i*_, *i* = *x*, *θ*, and *z*, are the stresses and strains in the axial (*x*), circumferential (*θ*), and radial (*z*) directions. *υ* and *E* are Poisson's ratio and modulus of elasticity, respectively. In (6), *λ* is

(7) $$\begin{array}{c} \lambda =\frac{E}{\left(1+\upsilon \right)\left(1-2\upsilon \right)}. \end{array}$$

The normal forces (*N*_*x*_, *N*_*θ*_, *N*_*z*_), bending moments (*M*_*x*_, *M*_*θ*_, *M*_*z*_), shear force (*Q*_*x*_), and the torsional moment (*M*_*xz*_) in terms of stress resultants are

(8) $$\begin{array}{c} \left\{\begin{array}{c} N_{x}\\ N_{\theta }\;\\ N_{z} \end{array}\right\}=\overset{h/2}{\underset{-h/2}{\int }}\left\{\begin{array}{c} \sigma_{x}\left(1+\frac{z}{R}\right)\\ \sigma_{\theta }\\ \sigma_{z}\left(1+\frac{z}{R}\right) \end{array}\right\}dz,\\ \left\{\begin{array}{c} M_{x}\\ M_{\theta }\;\\ M_{z} \end{array}\right\}=\overset{h/2}{\underset{-h/2}{\int }}\left\{\begin{array}{c} \sigma_{x}\left(1+\frac{z}{R}\right)\\ \sigma_{\theta }\\ \sigma_{z}\left(1+\frac{z}{R}\right) \end{array}\right\}zdz,\\ Q_{x}=k\overset{h/2}{\underset{-h/2}{\int }}\tau_{xz}\left(1+\frac{z}{R}\right)dz,\\ M_{xz}=K\overset{h/2}{\underset{-h/2}{\int }}\tau_{xz}\left(1+\frac{z}{R}\right)zdz, \end{array}$$

where *k* is the shear correction factor that is embedded in the shear stress term. In the static state, for cylindrical shells, *k* = 5/6 [16].

On the basis of the principle of virtual work, the variations of strain energy are equal to the variations of work of external forces as follows:

(9) $$\begin{array}{c} \delta U=\delta W, \end{array}$$

where *U* is the total strain energy of the elastic body and *W* is the total work of external forces due to internal pressure and centrifugal force.

With substituting strain energy and work of external forces, we have [8]

(10) ∫0LR(x)∫−h/2h/2(σxδεx+σθδεθ+σzδεz+τxyδγxz)     ×(1+zR)dz dx =∫0LPδUz(R−h2)dx−ρω2  ×∫0L∫−h/2h/2(R+z)2δUzdz dx,

where *ρ* is the density and *ω* is the constant angular velocity. *ρω* ^2^ is the force per unit volume due to centrifugal force. Substituting (5) and (6) into (10), and drawing upon calculus of variation and the virtual work principle, we will have

(11) NxR=C0,MxdRdx+R(dMxdx−Qx)=0,QxdRdx+R(dQxdx)−Nθ =−P(R−h2)−ρω26h2(12R2+h2),MxzdRdx+R(dMxzdx−Nz)−Mθ =Ph2(R−h2)−ρω26Rh3.

And the boundary conditions are

(12) $$\begin{array}{c} {\left[\left(N_{x}\delta u+M_{x}\delta \phi +Q_{x}\delta w+M_{xz}\delta \psi \right)R\right]}_{0}^{L}=0. \end{array}$$

Equation (12) states the boundary conditions which must exist at the two ends of the cylinder.

In order to solve the set of differential equations (11), with use of (5) to (8) and then using (11), we have

(13) $$\begin{array}{c} \left[B_{1}\right]\frac{d^{2}}{dx^{2}}\left\{y\right\}+\left[B_{2}\right]\frac{d}{dx}\left\{y\right\}+\left[B_{3}\right]\left\{y\right\}=\left\{F\right\},\\ \left\{y\right\}={\left\{\begin{array}{cccc} \frac{du}{dx} & \phi & w & \psi \end{array}\right\}}^{T}. \end{array}$$

The coefficients matrices [*B*_*i*_]_4×4_ and force vector {*F*}_4×1_ are as follows:

(14) $$\begin{array}{c} \left[B_{1}\right]=\left[\begin{array}{cccc} 0 & 0 & 0 & 0\\ 0 & \left(1-\upsilon \right)\frac{h^{3}}{12}R & 0 & 0\\ 0 & 0 & \mu hR & \frac{\mu h^{3}}{12}\\ 0 & 0 & \frac{\mu h^{3}}{12} & \frac{\mu h^{3}}{12}R \end{array}\right],\\ \left[B_{2}\right]=\left[\begin{array}{cccc} 0 & \left(1-\upsilon \right)\frac{h^{3}}{12} & 0 & 0\\ \left(1-\upsilon \right)\frac{h^{3}}{12} & \left(1-\upsilon \right)\frac{h^{2}}{12}\left(3R\frac{dh}{dx}+h\frac{dR}{dx}\right) & -\mu hR & -\left(\mu -2\upsilon \right)\frac{h^{3}}{12}\\ 0 & \mu hR & \mu \left(R\frac{dh}{dx}+h\frac{dR}{dx}\right) & \frac{\mu h^{2}}{4}\frac{dh}{dx}\\ 0 & \left(\mu -2\upsilon \right)\frac{h^{3}}{12} & \frac{\mu h^{2}}{4}\frac{dh}{dx} & \frac{\mu h^{2}}{12}\left(3R\frac{dh}{dx}+h\frac{dR}{dx}\right) \end{array}\right],\\ \left[B_{3}\right]=\left[\begin{array}{cccc} \left(1-\upsilon \right)hR & 0 & \upsilon h & \upsilon hR\\ \left(1-\upsilon \right)\frac{h^{2}}{4}\frac{dh}{dx} & -\mu hR & 0 & \frac{vh^{2}}{2}\frac{dh}{dx}\\ -\upsilon h & \mu \left(R\frac{dh}{dx}+h\frac{dR}{dx}\right) & -\left(1-\upsilon \right)\alpha & -h+\left(1-\upsilon \right)\alpha R\\ -\upsilon hR & \frac{\mu h^{2}}{4}\frac{dh}{dx} & -h+\left(1-\upsilon \right)\alpha R & -\left(1-\upsilon \right)\alpha R^{2} \end{array}\right],\\ \left\{F\right\}=\frac{1}{\lambda }\left\{\begin{array}{c} C_{0}\\ 0\\ -P\left(R-\frac{h}{2}\right)-\frac{\rho \omega^{2}}{6}\frac{h}{2}\left(12R^{2}+h^{2}\right)\\ P\frac{h}{2}\left(R-\frac{h}{2}\right)-\frac{\rho \omega^{2}}{6}Rh^{3} \end{array}\right\}, \end{array}$$

where the parameters are as follows:

(15) μ=512(1−2υ),α=ln⁡⁡(R+h/2R−h/2).

The set of differential equations (13) is solved by perturbation technique in [8]. In the next section, a new method is presented for solving set of (11).

## 3. Solution with Disk Form Multilayers

**Figure 2:**
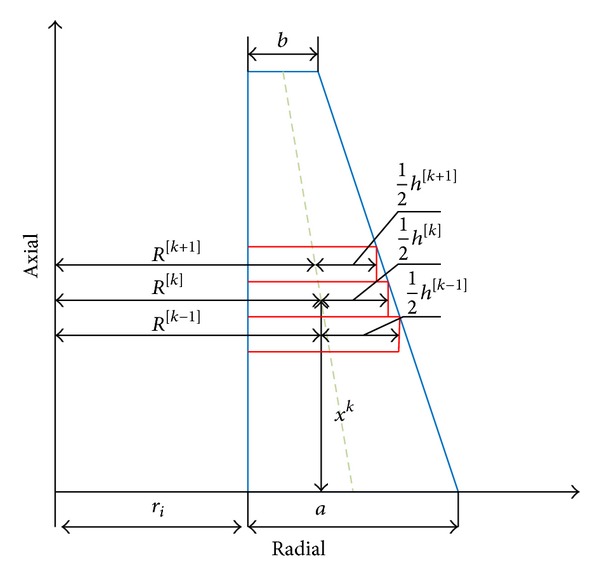
Dividing of thick cylinder with variable thickness to disk form multilayers.

In this method, the thick cylinder with variable thickness is divided into disk layers with constant height *h* (Figure 2).

Therefore, the governing equations convert to nonhomogeneous set of differential equations with constant coefficients. *x* ^[*k*]^ and *R* ^[*k*]^ are length and radius of middle of disks. *k* is number of disks. The modulus of elasticity and Poisson's ratio of disks are assumed be constant.

**Figure 3:**
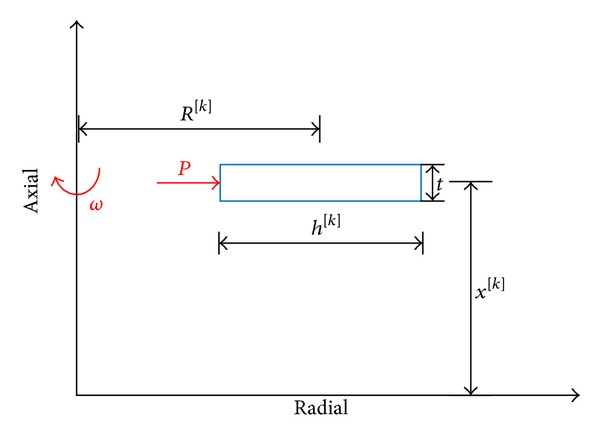
Geometry of an arbitrary disk layer.

The length of middle of an arbitrary disk (Figure 3) is as follows:

(16) $$\begin{array}{c} x^{\left[k\right]}=\left(k-\frac{1}{2}\right)\frac{L}{n},\\ \left(x^{\left[k\right]}-\frac{t}{2}\right)\leq x\leq \left(x^{\left[k\right]}+\frac{t}{2}\right),\\ t=\frac{L}{n}, \end{array}$$

where *n* is the number of disks and *k* is the corresponding number given to each disk.

The radius of middle point of each disk is as follows:

(17) $$\begin{array}{c} R^{\left[k\right]}=r_{i}+\frac{h^{\left[k\right]}}{2},\\ h^{\left[k\right]}=a-\tan\left(\beta \right)x^{\left[k\right]}. \end{array}$$

Thus,

(18) (dhdx)[k]=2(dRdx)[k]=−tan⁡β.

Considering shear stress and based on FSDT, nonhomogeneous set of ordinary differential equations with constant coefficient of each disk is obtained:

(19) $$\begin{array}{c} {\left[B_{1}\right]}^{\left[k\right]}\frac{d^{2}}{{dx}^{2}}{\left\{y\right\}}^{\left[k\right]}+{\left[B_{2}\right]}^{\left[k\right]}\frac{d}{dx}{\left\{y\right\}}^{\left[k\right]}+{\left[B_{3}\right]}^{\left[k\right]}{\left\{y\right\}}^{\left[k\right]}={\left\{F\right\}}^{\left[k\right]},\\ {\left\{y\right\}}^{\left[k\right]}={\left\{\begin{array}{cccc} {\left(\frac{du}{dx}\right)}^{\left[k\right]} & \phi^{\left[k\right]} & w^{\left[k\right]} & \psi^{\left[k\right]} \end{array}\right\}}^{T}. \end{array}$$

The coefficients matrices [*B*_*i*_]_4×4_^[*k*]^ and force vector {*F*}_4×1_^*k*^ are as follows:

(20) [B1][k]=[00000(1−υ)(h[k])312R[k]0000μh[k]R[k]μ(h[k])31200μ(h[k])312μ(h[k])312R[k]],[B2][k]=[0(1−υ)(h[k])31200(1−υ)(h[k])312−(1−υ)(h[k])224(6R[k]+h[k])tan⁡β−μh[k]R[k]−(μ−2υ)(h[k])3120μh[k]R[k]−μ(R[k]+h[k]2)tan⁡β−μ(h[k])24tan⁡β0(μ−2υ)(h[k])312−μ(h[k])24tan⁡β−μ(h[k])224(6R[k]+h[k])tan⁡β],[B3][k]=[(1−υ)h[k]R[k]0υh[k]υh[k]R[k]−(1−υ)(h[k])24tan⁡β−μh[k]R[k]0−υ(h[k])22tan⁡β−υh[k]−μ(R[k]+h[k]2)tan⁡β−(1−υ)α[k]−h[k]+(1−υ)α[k]R[k]−υh[k]R[k]−μ(h[k])24tan⁡β−h[k]+(1−υ)α[k]R[k]−(1−υ)α[k](R[k])2],{F}[k]=1λ{C00−P(R[k]−h[k]2)−ρω26h[k]2(12(R[k])+(h[k])2)Ph[k]2(R[k]−h[k]2)−ρω26(h[k])3R[k]},

where the parameters are as follows:

(21) $$\begin{array}{c} \mu =\frac{5}{12}\left(1-2\upsilon \right),\\ \alpha^{\left[k\right]}=\ln\left(\frac{R^{\left[k\right]}+h^{\left[k\right]}/2}{R^{\left[k\right]}-h^{\left[k\right]}/2}\right). \end{array}$$

Defining the differential operator *P*(*D*), (19) is written as

(22) $$\begin{array}{c} {\left[P\left(d\right)\right]}^{\left[k\right]}={\left[B_{1}\right]}^{\left[k\right]}D^{2}+{\left[B_{2}\right]}^{\left[k\right]}D+{\left[B_{3}\right]}^{\left[k\right]},\\ D^{2}=\frac{d^{2}}{{dx}^{2}},\;\;D=\frac{d}{dx}. \end{array}$$

Thus

(23) [P(D)][k]{y}[k]={F}[k].

The above differential equation has the total solution including general solution for homogeneous case {*y*}_*h*_^[*k*]^ and particular solution {*y*}_*P*_^[*k*]^ as follows:

(24) {y}[k]={y}h[k]+{y}P[k].

For the general solution for homogeneous case, {y}h[k]={V}[k]em[k]x is substituted in [*P*(*D*)]^[*k*]^{*y*}^[*k*]^ = 0.

We have

(25) $$\begin{array}{c} \left|m^{2}{\left[B_{1}\right]}^{\left[k\right]}+m{\left[B_{2}\right]}^{\left[k\right]}+{\left[B_{3}\right]}^{\left[k\right]}\right|=0. \end{array}$$

Thus

(26) |B11B12B13B14B21B22B23B24B31B32B33B34B41B42B43B44|=0,B11=(1−υ)h[k]R[k],B12=m(1−υ)(h[k])312,B13=−B31=υh[k],B14=−B41=υh[k]R[k],B21=(1−υ)(h[k])212(mh[k]−3tanβ),B22=(1−υ)(h[k])224[2m2R[k]h[k]−m(6R[k]+h[k])tanβ]−μh[k]R[k],B23=−mμh[k]R[k],B24=−(h[k])212[m(μ−2υ)h[k]+6υtanβ],B32=μ[mh[k]R[k]−(R[k]+h[k]2)tanβ],B33=μ[m2h[k]R[k]−m(R[k]+h[k]2)tanβ]−(1−υ)α[k],B34=B43=μ(h[k])212[m2(h[k])−3mtanβ]−(h[k]−(1−υ)α[k]R[k]),B42=(h[k])212(m(μ−2υ)h[k]−3μtanβ),B44=μ(h[k])224[2m2h[k]R[k]−m(6R[k]+h[k])tanβ]−(1−υ)α[k](R[k])2.

The result of the determinant above is a six-order polynomial which is a function of *m*, the solution of which is 6 eigenvalues *m*_*i*_. The eigenvalues are 3 pairs of conjugated root. Substituting the calculated eigenvalues in the following equation, the corresponding eigenvectors {*V*}_*i*_ are obtained as follows:

(27) $$\begin{array}{c} \left[m^{2}{\left[B_{1}\right]}^{\left[k\right]}+m{\left[B_{2}\right]}^{\left[k\right]}+{\left[B_{3}\right]}^{\left[k\right]}\right]{\left\{V\right\}}^{\left[k\right]}=0. \end{array}$$

Therefore, the homogeneous solution for (23) is

(28) $$\begin{array}{c} {\left\{y\right\}}_{h}^{\left[k\right]}=\overset{6}{\underset{i=1}{\sum }}C_{i}^{\left[k\right]}{\left\{V\right\}}_{i}^{\left[k\right]}e^{m_{i}^{\left[k\right]}x}. \end{array}$$

The particular solution is obtained as follows:

(29) $$\begin{array}{c} {\left\{y\right\}}_{p}^{\left[k\right]}={\left[{\left[B_{3}\right]}^{\left[k\right]}\right]}^{-1}{\left\{F\right\}}^{\left[k\right]}. \end{array}$$

Therefore, the total solution for (23) is

(30) $$\begin{array}{c} {\left\{y\right\}}_{}^{\left[k\right]}=\overset{6}{\underset{i=1}{\sum }}C_{i}^{}{\left\{V\right\}}_{i}^{\left[k\right]}e^{m_{i}^{\left[k\right]}x}+{\left[{\left[B_{3}\right]}^{\left[k\right]}\right]}^{-1}{\left\{F\right\}}^{\left[k\right]}. \end{array}$$

In general, the problem for each disk consists of 8 unknown values of *C*_*i*_, including *C*_0_ (first equation (11)), *C*_1_ to *C*_6_ (30), and *C*_7_ (Equation *u* ^[*k*]^ = ∫(*du*/*dx*)^[*k*]^ *dx* + *C*_7_).

## 4. Boundary and Continuity Conditions

In this problem, the boundary conditions of cylinder are clamped-clamped ends; then we have

(31) $$\begin{array}{c} {\left\{\begin{array}{c} u\\ \phi \\ w\\ \psi \end{array}\right\}}_{x=0}={\left\{\begin{array}{c} u\\ \phi \\ w\\ \psi \end{array}\right\}}_{x=L}=\left\{\begin{array}{c} 0\\ 0\\ 0\\ 0 \end{array}\right\}. \end{array}$$

Therefore,

(32) $$\begin{array}{c} {\left\{\begin{array}{c} U_{x}\left(x,z\right)\\ U_{z}\left(x,z\right) \end{array}\right\}}_{x=0,L}=\left\{\begin{array}{c} 0\\ 0 \end{array}\right\}. \end{array}$$

Because of continuity and homogeneity of the cylinder, at the boundary between two layers, forces, stresses and displacements must be continuous. Given that shear deformation theory applied is an approximation of one order and also all equations related to the stresses include the first derivatives of displacement, the continuity conditions are as follows:

(33) $$\begin{array}{c} {\left\{\begin{array}{c} U_{x}^{\left[k-1\right]}\left(x,z\right)\\ U_{z}^{\left[k-1\right]}\left(x,z\right) \end{array}\right\}}_{x=x^{\left[k-1\right]}+t/2}={\left\{\begin{array}{c} U_{x}^{\left[k\right]}\left(x,z\right)\\ U_{z}^{\left[k\right]}\left(x,z\right) \end{array}\right\}}_{x=x^{\left[k\right]}-t/2},\\ {\left\{\begin{array}{c} U_{x}^{\left[k\right]}\left(x,z\right)\\ U_{z}^{\left[k\right]}\left(x,z\right) \end{array}\right\}}_{x=x^{\left[k\right]}+t/2}={\left\{\begin{array}{c} U_{x}^{\left[k+1\right]}\left(x,z\right)\\ U_{z}^{\left[k+1\right]}\left(x,z\right) \end{array}\right\}}_{x=x^{\left[k+1\right]}-t/2},\\ {\left\{\begin{array}{c} \frac{dU_{x}^{\left[k-1\right]}\left(x,z\right)}{dx}\\ \frac{dU_{z}^{\left[k-1\right]}\left(x,z\right)}{dx} \end{array}\right\}}_{x=x^{\left[k-1\right]}+t/2}={\left\{\begin{array}{c} \frac{dU_{x}^{\left[k\right]}\left(x,z\right)}{dx}\\ \frac{dU_{z}^{\left[k\right]}\left(x,z\right)}{dx} \end{array}\right\}}_{x=x^{\left[k\right]}-t/2},\\ {\left\{\begin{array}{c} \frac{dU_{x}^{\left[k\right]}\left(x,z\right)}{dx}\\ \frac{dU_{z}^{\left[k\right]}\left(x,z\right)}{dx} \end{array}\right\}}_{x=x^{\left[k\right]}+t/2}={\left\{\begin{array}{c} \frac{dU_{x}^{\left[k+1\right]}\left(x,z\right)}{dx}\\ \frac{dU_{z}^{\left[k+1\right]}\left(x,z\right)}{dx} \end{array}\right\}}_{x=x^{\left[k+1\right]}-t/2}. \end{array}$$

Given the continuity conditions, in terms of *z*, 8 equations are obtained. In general, if the cylinder is divided into *n* disk layers, 8(*n* − 1) equations are obtained. Using the 8 equations of boundary condition, 8*n* equations are obtained. The solution of these equations yields 8*n* unknown constants.

## 5. Results and Discussion

A cylindrical shell with *r*_*i*_ = 40 mm, *a* = 20 mm, *b* = 10 mm, and *L* = 800 mm will be considered in this paper. For analytical and numerical results the properties used are *E* = 200 GPa and *υ* = 0.3. The applied internal pressure is 80 MPa. The thick cylindrical shell with variable thickness has clamped-clamped boundary conditions.

**Figure 4:**
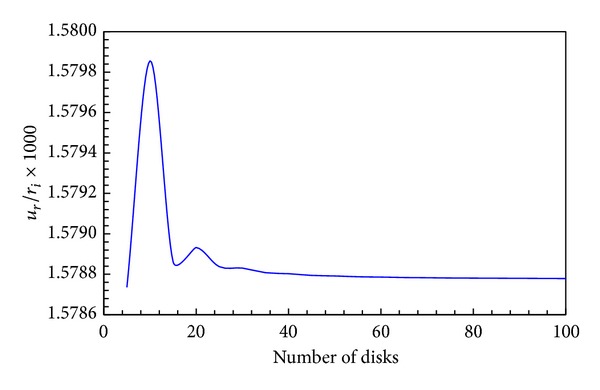
Effect of the number of disk layers on the radial displacement.

The effect of the number of disk layers on the radial displacement is shown in Figure 4. It is observed that if the number of disk layers is less than 50, it will have a significant effect on the response. However, if the number of layers is more than 60 disks, there will be no significant effect on radial displacement. In the problem in question 75 disks are used.

**Figure 5:**
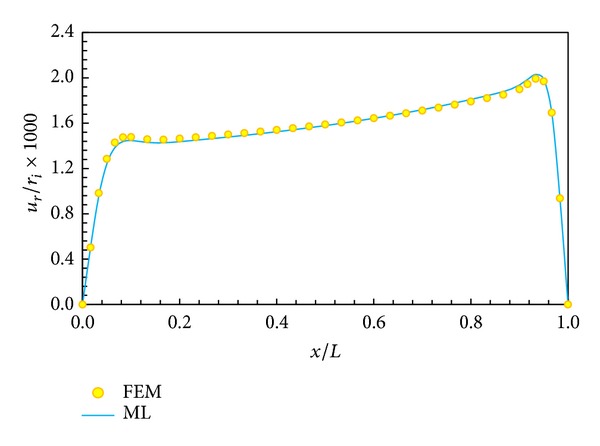
Radial displacement distribution in middle layer.

**Figure 6:**
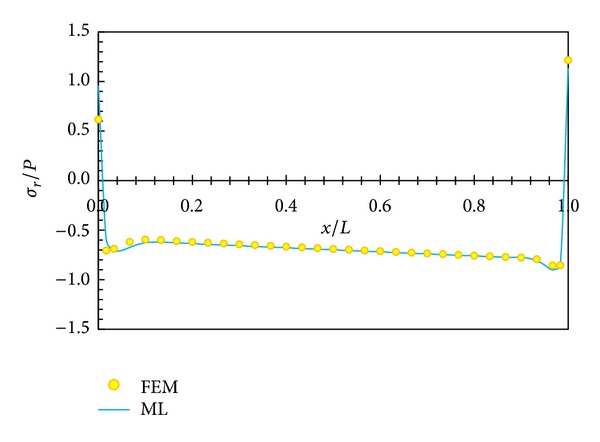
Radial stress distribution in middle layer.

**Figure 7:**
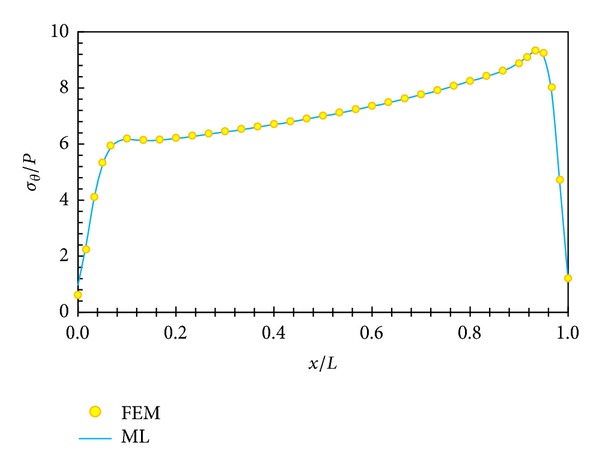
Circumferential stress distribution in middle layer.

**Figure 8:**
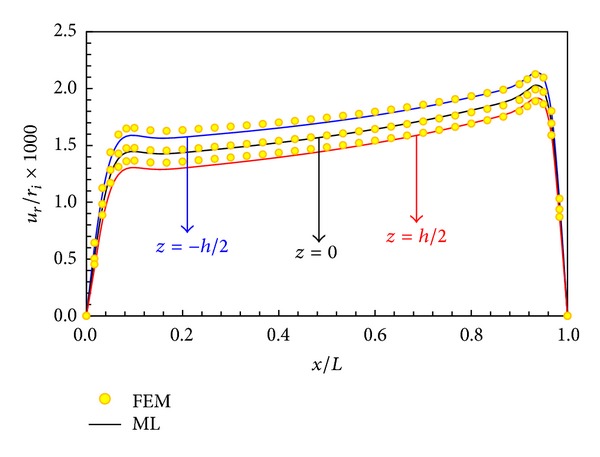
Radial displacement distribution in different layers.

In Figures 5, 6, 7, and 8, displacement and stress distributions are obtained using multilayer method (ML), are compared with the solutions of FEM, and are presented in the form of graphs. Figures 5 to 8 show that the disk layer method based on FSDT has an acceptable amount of accuracy when one wants to obtain radial displacement, radial stress, circumferential stress, and shear stress.

The distribution of radial displacement at different layers is plotted in Figure 8. The radial displacement at points away from the boundaries depends on radius and length. According to Figure 8 the change in radial displacements in the lower boundary is greater than that of the upper boundary and the greatest radial displacement occurs in the internal surface (*z* = −*h*/2).

**Figure 9:**
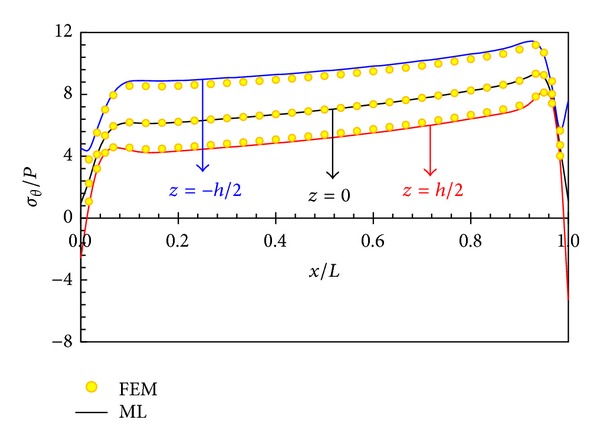
Circumferential stress distribution in different layers.

Distribution of circumferential stress in different layers is shown in Figure 9. The circumferential stress at all points depends on radius and length. The circumferential stress at layers close to the external surface is negative and at other layers positive. The greatest circumferential stress occurs in the internal surface.

**Figure 10:**
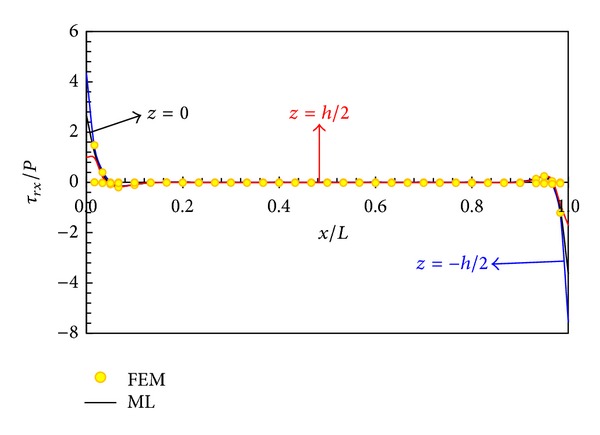
Shear stress distribution in different layers.

Figure 10 shows the distribution of shear stress at different layers. The shear stress at points away from the boundaries at different layers is the same and trivial. However, at points near the boundaries, the stress is significant, especially in the internal surface, which is the greatest.

**Figure 11:**
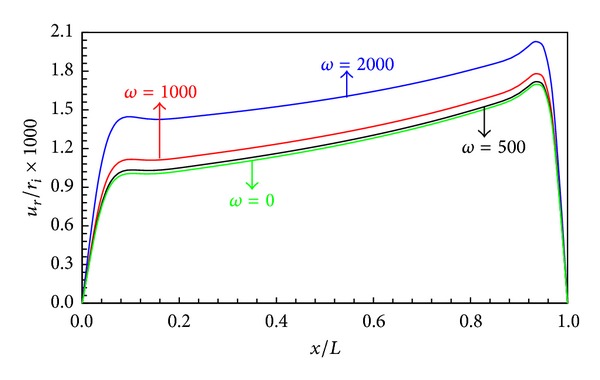
Radial displacement distribution in middle layers.

**Figure 12:**
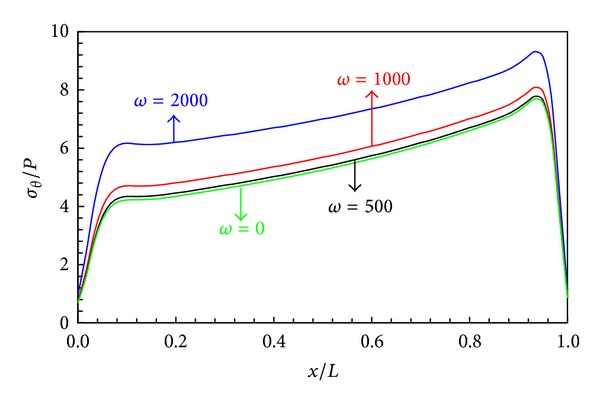
Circumferential stress distribution in middle layers.

**Figure 13:**
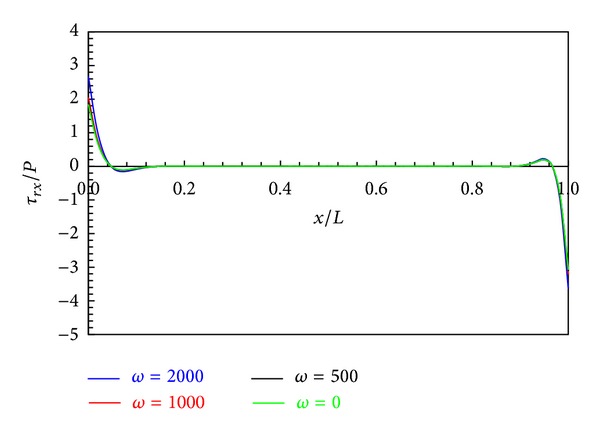
Shear stress distribution in middle layers.

The effects of angular velocity *ω* on the distribution of the stresses and radial displacement are presented in Figures 11–13.

Figures 11 and 12 indicate radial displacement and circumferential stress rise with increasing angular velocity. Also for the angular speed less than 500 rad/s, the centrifugal force is less effective than the internal pressure.

According to Figure 13, the shear stress is independent of the centrifugal force. Also, it is noted that the shear stress at points away from the boundaries is zero.

## 6. Conclusions

In the present study, we have the following.

1. Based on FSDT and elasticity theory, the governing equations of thick-walled disks are derived.
2. A thick cylindrical shell with variable thickness is divided into disks with constant height.
3. With considering continuity between layers and applying boundary conditions, the governing set of differential equations with constant coefficients is solved.
4. The results obtained for stresses and displacements are compared with the solutions carried out through the FEM. Good agreement was found among the results.

Adventures of the semi-analytical using disk form multilayers are as follows.

- First shear deformation theory and perturbation theory result in the analytical solution of the problem with higher accuracy and within a shorter period of time.
- The solutions are complicated and time consuming.
- The shells with different geometries, and different loadings, and different boundary conditions, with even variable pressure, could be more easily solved.
- The method is very suitable for the purpose of calculation of radial stress, circumferential stress, shear stress, and radial displacement.

Finally, in spite of the existing analytical methods, due to their complex mathematical relations governing them, could not easily solve them. Therefore, the multilayer disk form method could be a good replacement for the analysis of thick-walled shells.
